# Sleep and inflammation in resilient aging

**DOI:** 10.1098/rsfs.2014.0009

**Published:** 2014-10-06

**Authors:** Michael R. Irwin

**Affiliations:** 1 Cousins Center for Psychoneuroimmunology, UCLA Semel Institute for Neuroscience, University of California, Los Angeles, CA, USA; 2 Department of Psychiatry and Biobehavioral Sciences, David Geffen School of Medicine, University of California, Los Angeles, CA, USA

**Keywords:** sleep, aging, resilience pain, depression, mortality, inflammation

## Abstract

Sleep quality is important to health, and increasingly viewed as critical in promoting successful, resilient aging. In this review, the interplay between sleep and mental and physical health is considered with a focus on the role of inflammation as a biological pathway that translates the effects of sleep on risk of depression, pain and chronic disease risk in aging. Given that sleep regulates inflammatory biologic mechanisms with effects on mental and physical health outcomes, the potential of interventions that target sleep to reduce inflammation and promote health in aging is also discussed.

## 1. Introduction

Recent years have seen increasing public health attention to the contribution of sleep quality to health. Furthermore, this relationship is especially salient in older adults who are at risk for sleep disturbance, as well as chronic diseases of aging. Indeed, in two separate reports from the Institute of Medicine, *Health and Behavior* [1,2], a range of research aimed at understanding the interplay among biological, behavioural and social factors in health and disease was identified and integrated. In this review, we consider the role of sleep in promoting healthy, resilient aging. First, we discuss the myriad effects of sleep on health and the associations between sleep disturbance and physical health outcomes. Second, given that subjective symptoms such as pain and depression often compromise quality of life in older adults and diminish successful aging, we consider the role of sleep in adaptation to the psychosocial and physical challenges of aging and the contribution of sleep and/or its disruption to pain perceptions and depressive symptoms. Third, we identify the reciprocal links between the central nervous system and the immune system, which together organize responses to infections and other challenges. Indeed, regulation of inflammatory biology dynamics appears to be a key biologic pathway that determines to what the degree an older adult is able to maintain health. Conversely, activation of inflammatory signalling can increase risk for disease, and also induce depressive symptoms, which together compromise resilient aging. Finally, because sleep regulates inflammatory biologic mechanisms, we consider the potential of interventions that target sleep to reduce inflammation and promote health in aging.

## 2. Definition of resilience

Resilience is a dynamic process whereby an individual exhibits adaptation in response to significant adversity [3]. Defined by psychological aspects of coping, as well as physiological recovery from stress, resilience reflects a two-way interaction between exposure to adversity and successful or positive recovery to that challenge. In addition, resilience is also defined as a successful adaptation to a condition that may be ongoing, which places the individual at risk for negative outcomes such as medical morbidity or depression. For example, successful aging represents a resilient response to the cumulative, additive and possibly exponential risks associated with old age. Conversely, when there is an absence of clinical symptoms, psychiatric distress or deterioration in health despite age-related physiological vulnerability and ongoing challenges to the social environment (e.g. loss of friends and family) and individual resources (e.g. health), an older adult is showing an adaptive response to the specific tasks of life consistent with successful aging. Importantly, risk factors may be cumulative, and carry additive effects, when they co-occur as found in aging [3]. In sum, resilience is conceptually characterized, along a continuum of adaptation or success, as an active response to either discrete adverse events, or to ongoing and cumulative conditions of psychobiological challenge including chronic medical conditions. In turn, resilience is also characterized by reduced chronic medical burden or the absence of disability due to such medical conditions. Resilience is different from strengths or assets, which are viewed as static characteristics.

## 3. Features of successful aging

When an individual reaches old age, accumulating negative conditions represent a serious challenge to the capacity to adapt and likely reduce the quality of life. Indeed, only 35.8% of older adults are defined as ‘successful agers’ [4]. The most frequent correlates of the various definitions of successful aging are age (young-old), non-smoking, absence of disability due to chronic medical disorders such as arthritis and cardiovascular disease, fewer medical disorders and absence of depression. By contrast, continuity of resilience into retirement years is unaffected, for example, by gender and education. Nevertheless, in a study of 205 community-dwelling elderly, 92% of the participants rated themselves as aging successfully [5], even in the presence of chronic medical illness, which suggests that individual characteristics rather than external events (e.g. comorbid illness, income) determine successful aging. However, little is known about the biobehavioural determinants of resilient aging, although multiple biological systems likely relate to the states of reduced risk of medical comorbidities, disability and depression.

## 4. Sleep and healthy aging

Restorative sleep is hypothesized to be one determinant of resilience to life challenges, with increasing evidence suggesting that adequate sleep amounts and sleep quality promote healthy, successful aging along physical (e.g. medical morbidity), psychological (e.g. depression and pain) and biological domains (e.g. inflammation). Conversely, sleep disturbance might be conceptualized as an adverse condition, which increases risk for chronic disease and depression in older adults. Indeed, as reviewed below, alterations of sleep wake activity have potent effects on health outcomes including domains of mortality, chronic disease morbidity, depression and hyperalgesia.

Poor sleep is one of the most common complaints in older adults. Even in healthy seniors, prevalence rates of insomnia exceed 20–30%, greater in frequency and severity than any other age group [6–8]. Indeed, normal aging is associated with declines in subjective sleep quality, sleep fragmentation, increases of light (stages 1 and 2) sleep and decreases of deep slow-wave sleep [9–12].

Adequate sleep amounts appear to promote lower rates of medical morbidity, with an optimal amount of sleep around 7 h per night. Indeed, more than 40 epidemiologic studies have demonstrated a U-shaped association of sleep duration with mortality, with the lowest mortality associated with approximately 7 h of self-reported sleep [13–18]. This association has been demonstrated in studies of more than 1 million participants, statistical control for up to 30 potential comorbidities and covariates, and follow-up durations as long as 10–20 years [19]. Similar patterns have been noted for men and women and all adult age strata, though older adults have shown the highest mortality rates, and the greatest excess mortality associated with short and long sleep.

In addition to mortality risk, similar U-shaped associations have been found between sleep duration and multiple morbidities, including heart disease [20], stroke [21], hypertension [22], diabetes [23], depression [18], obesity and metabolic syndrome [24]. As reviewed below, short and long sleep are associated with inflammation [25], which might contribute to these morbidities. Of course, epidemiologic data cannot fully prove that short or long sleep causes mortality/morbidity. If short or long sleep truly causes morbidity—which could only be tested with controlled trials—then modest changes in sleep duration could have a significant impact on health and possibly mortality. Indeed, short or long sleep would be the fifth leading cause of death in the USA [26] were short or long sleep duration to *cause* all the deaths with which it has been associated in epidemiologic studies.

Furthermore, the absence of sleep complaints (i.e. when sleep is reported as adequate and restorative) might also have protective effects on health, given naturalistic observations that difficulties with sleep have converse health implications. For example, epidemiological data have found that self-reported difficulties with sleep maintenance (i.e. insomnia) act as a prospective predictor of cardiovascular and non-cardiovascular disease mortality, particularly in community elderly populations [6,13,20,27–32]. Furthermore, electroencephalogram measures confirm that older adults who routinely take longer than 30 min to fall asleep have higher rates of mortality as compared with those with short sleep latency; this association is found over and above the contribution of other known factors (e.g. age, gender and medical burden) in older adults (odds ratio (OR) 1.7) [28].

## 5. Sleep and depression risk in aging

Good sleep quality, as defined by self-reported sleep that is restorative and maintained throughout the night, also appears to be protective of mental health in older adults. A recent meta-analysis of longitudinal epidemiologic studies found that self-reported insomnia substantially increases the risk of depression. Indeed, insomnia symptoms increased the depression risk by over twofold (OR: 2.6, confidence interval: 1.98–3.42), although results were heterogeneous [33]. However, findings in older adults are limited, with only six studies identified in this age group, including clinical and primary care populations [34–39]. Furthermore, only two prospective studies describe the effect of sleep disturbance on depression risk in community-dwelling older adults [34,38], with some evidence that the effects of sleep disturbance on depression is only found in those with a prior history of depression [34] or when sleep disturbance is persistent [40]. Indeed, sleep disturbance is more robustly related with depression recurrence, as opposed to its new incidence, in older adults [34,40].

## 6. Sleep and pain

Pain is also a common complaint that contributes to disability in older adults. However, little is known about whether adequate sleep might diminish hyperalgesia in those at risk for chronic pain such as older adults with chronic medical conditions such as osteroarthritis or patients with rheumatoid arthritis. Nevertheless, observational and experimental studies suggest that sleep and/or its disruption contribute to hyperalgesia. For example, cross-sectional studies in rheumatoid arthritis patients have found that sleep disturbance correlates with greater pain and disease activity [41,42], which indicates increased medical burden. Additionally, polysomnographic studies show that chronic pain is associated with poor sleep continuity and reduced total sleep time in other populations [43].

Whereas it is often thought that difficulties with sleep are due to pain, sleep disturbance and pain appear to be bidirectionally related. The occurrence of pain at night can disrupt sleep, but it also is possible that sleep disturbance can drive increased pain perception in the day after sleep. Whereas data are constrained by small sample sizes, some experimental evidence shows that sleep loss increases pain reporting [44–49]. In some instances, however, assessment of pain has relied on self-reports without inclusion of comparison controls, raising the possibility of confounding reporting bias. Furthermore, many of the findings linking sleep disturbance to pain perception may not be generalizable to the clinical populations, as the manipulation selectively targeted a particular sleep stage (e.g. rapid eye movement (REM) sleep) or used total sleep deprivation. Most clinical populations, in contrast, evidence sleep loss during part of the night or experience sleep fragmentation, which may not generate the same response as either loss of a sleep stage or sleep throughout the entire night. Nevertheless, two studies have found that selective slow-wave sleep deprivation decreased mechanical pain thresholds [45,46], and one study found that REM sleep deprivation increased thermal pain sensitivity [48]. Similarly, total sleep deprivation reportedly induces hyperalgesia or enhanced responsivity to painful stimuli. Moreover, when sleep deprivation occurs over two nights, increases of pain are even greater than after a single night, suggesting a dose–response relationship [44,47,49]. Experimental fragmentation with disruption of sleep continuity also has been found to induce spontaneous pain similar to sleep restriction [50].

Recently, we have extended these observations and evaluated the resilient responses of rheumatoid arthritis versus comparison controls to the challenge of sleep loss [51]. In these studies, it was hypothesized that the at-risk population of rheumatoid arthritis patients will show a failure to adapt to sleep loss, which will lead to relatively greater and more persistent elevations of pain. Consistent with this hypothesis, rheumatoid patients appear to be less resilient to the challenge of sleep loss. As compared with controls, rheumatoid arthritis patients show an exaggerated increase in symptoms of pain after sleep loss. Furthermore, there is an activation of disease-specific pain in rheumatoid arthritis patients in which sleep loss was found to induce hyperalgesia, which was accompanied by increases in the number of painful joints and the severity of associated joint pain. Importantly, independent clinician rating of painful and tender joints confirmed increases in disease-specific pain following sleep loss in rheumatoid arthritis patients.

Sleep loss is also known to induce feelings of depression and fatigue, which may co-occur or possibly exacerbate pain perceptions given cross-sectional and prospective models showing that depression, fatigue and pain are related. Again, rheumatoid arthritis patients who face ongoing difficulties with pain and sleep disturbance may be more vulnerable or less resilient to the effects of sleep loss, which would lead to relatively greater increases in self-reported symptoms of depression and fatigue. Indeed, rheumatoid arthritis patients have higher levels of depressed mood and fatigue at baseline and show greater and more persistent increases of these symptoms after sleep loss [51].

## 7. Inflammation and aging

Increasing evidence also implicates inflammation as being a key biologic pathway that determines to what degree an older adult becomes vulnerable to chronic disease or maintains health in a model of resilient aging. In general, aging is associated with an increased expression of proinflammatory markers [52,53]. However, even though inflammation is found in aggregate with aging, such activation of inflammatory signals is not universal. Indeed, only a fraction of older adults appear to show increases in inflammation, with some studies reporting that less than 25–30% of older adults show elevated levels of systemic inflammation (e.g. C-reactive protein, CRP) as defined by clinical threshold criteria. Yet, among these older adults with elevated levels of inflammatory cytokines, the risk for disability, morbidity and mortality is greatest [54]. For example, increases of the pleiotropic cytokine interleukin-6 (IL-6) have been found to influence the onset and course of a wide spectrum of age-associated diseases including cardiovascular disease, arthritis, type 2 diabetes and certain cancers [52]. In older adults, such elevated levels of IL-6 prospectively predict future disability, declines of health status and mortality risk [55,56]. The mechanisms that contribute to inflammation during aging are not fully understood, although substantial data indicate that aging impacts the efficiency of nuclear factor (NF)-κB signalling. The NF-κB transcription control pathway plays a key role in controlling cellular expression of proinflammatory genes and activation of this signalling pathway is implicated in the pathophysiology of diseases such as cardiovascular disease and cancer [57].

## 8. Inflammatory biology and mental health in older adults

In addition to the effects of inflammatory biology on disease risk, there is substantial evidence that levels of inflammatory activation contribute to behavioural symptoms [58], which might be related to the risk of depression in older adults. In other words, adults who maintain low levels of inflammation into older age may be more resilient to the challenges of aging (i.e. bereavement, sleep disturbance and disability) and may be less likely to have the onset of a depressive disorder [59]. Conversely, older adults who show activation of inflammatory signalling may be more likely to evidence symptoms of fatigue and depression, for example.

Peripheral proinflammatory signals are known to act on the brain through key behaviour-modulating multiple neurotransmitter systems including norepinephrine, dopamine and serotonin [60]. Such activation of brain neurotransmitter systems in turn activates a number of physiological and behavioural responses, which are generally conceptualized as sickness behaviours. In our studies, we have experimentally examined the impact of inflammatory challenge on behavioural responses by administering a low dose of endotoxin that induces a rapid and transient increase in a number of proinflammatory cytokines including IL-6 and tumour necrosis factor (TNF). Experimentally induced increases in proinflammatory cytokines influence the social or affective neural processes that induce depressed mood [61,62]. Furthermore, biological variability in the inflammatory response, as measured by circulating concentrations of IL-6, is important in relating to differences in neural responses from the experimental challenge itself, in which greater IL-6 increases were associated with greater activity in the anterior and posterior insula, regions involved in both physical and social pain processing, and activity in both of these regions correlated with increases in depressed mood. Interestingly, males appeared to be more resilient to the behavioural and neural effects of the inflammatory challenge as compared with females. [61]. In addition to inducing greater increases in self-reported and observer-rated depressed mood, inflammation diminishes the experience of pleasure as indexed by significant reductions in ventral striatum activity to reward cues [63].

## 9. Sleep and inflammation

Sleep quality may buffer age-related increases in inflammation. For example, greater sleep efficiency and more positive social relations predicted lower levels of the inflammatory marker IL-6, whereas poor sleep efficiency and poor social relations were associated with higher levels of IL-6 [64]. These findings indicate that sleep quality and social support interact to predict risk of inflammation or that both good sleep quality and adequate social support are protective against age-related increases of inflammation.

Sleep amounts and sleep architecture also play a substantial role in structuring overall inflammatory homeostasis. Partial sleep loss is ubiquitous in clinical populations, as well as older adults, and experimentally induced partial sleep deprivation may impact the homeostasis of proinflammatory cytokine activity. In contrast to adequate sleep amounts averaging 7–8 h in duration, sleep loss induces increases in cellular and genomic markers of inflammation. For example, partial sleep deprivation increases circulating levels of inflammatory markers, such as IL-6, TNF-α and CRP [65–67], with significant elevations after only one night of sleep loss [67]. Additional studies have examined the functional basis for altered inflammatory response after sleep loss by measuring the production of proinflammatory cytokines by monocytes following ligation of the toll-like receptor 4 (TLR4) with lipopolysaccharide [68]. Such cellular production of IL-6 and TNF due to aberrant increases of TLR activity has been linked to inflammatory diseases such as rheumatoid arthritis [69] and heart failure [70]. In the morning after a night of sleep loss, monocyte production of IL-6 and TNF-α was significantly greater relative to morning levels following uninterrupted sleep. In addition, sleep loss induced a more than threefold increase in transcription of IL-6 mRNA and a twofold increase in TNF-α mRNA [68]. The NF-κB transcription control pathway plays a key role in the inflammatory signalling cascade, and sleep loss also contributes to NF-κB activation [71]. Moreover, females appear to be especially vulnerable to such activation of inflammatory signalling processes, with greater increases in activation of NF-κB as well as the cellular production of IL-6 and TNF [71,72]. Activation of inflammatory biology also appears to constitute one element of a more general genomic response to sleep deprivation. Sleep deprivation induces an upregulation of a gene ensemble that includes the master circadian regulator, several ‘immediate early genes’ marking cellular signal transduction and multiple inflammatory response genes. Transcription factor-binding motifs that were over-represented in the sleep deprivation condition included the promoters of genes involved in regulation by cAMP/PKA-induced transcription factors of the CREB/ATF family, the PKC-induced AP-1 family, the proinflammatory NF-κB/Rel family and the MAP kinase-inducible ETS transcription factor family typified by ELK1. In sum, the leucocyte transcriptional response to sleep deprivation involves multiple signal transduction pathways including the NF-κB inflammatory signalling system.

## 10. Interventions to promote healthy sleep: relation to inflammation and well-being in aging

Among older adults, the use of behavioural approaches for the treatment of insomnia is increasingly viewed as an effective and recommended alternative to pharmacologic therapies, given that behavioural treatment can be administered without the risk of side effects found with pharmacotherapy [73]. Three recent meta-analyses support the efficacy of these behavioural approaches [73–75], and one comparative meta-analysis found that behaviour therapy and pharmacologic treatments yielded similar improvements in sleep maintenance, total sleep time and sleep quality, with some advantage for behaviour treatments in improving sleep latency [76]. Importantly, gains from various behavioural treatments have been demonstrated across all sleep outcomes with the exception of total sleep time, with evidence from a few studies in older adults that these benefits are sustained for months to years following treatment.

The strategies used in behavioural treatments are heterogeneous and include various approaches ranging from relaxation; behavioural focused treatments such as stimulus control and sleep restriction; and multi-component cognitive behavioural therapy that incorporates cognitive and behavioural targets along with sleep hygiene and possibly relaxation strategies. Despite the heterogeneity of these behavioural treatments, gains from these various approaches, as compared with placebo, do not differ substantially [74,75] as further reported in a recent meta-analysis [77].

Given that adults with insomnia symptoms are at substantial risk for developing syndromal insomnia, preventive treatments are also needed that target moderate sleep complaints prior to the onset of insomnia. Indeed, there is some evidence that physical exercise may enhance sleep quality, and King *et al*. [78] demonstrated that 16 weeks of community-based exercise training (i.e. endurance training, brisk walking and stationary cycling) was superior to a wait-listed control condition on measures of sleep quality in older adults with sub-syndromal insomnia. In addition, recent interest has focused on the use of a movement meditation, Tai Chi, to target insomnia symptoms. For example, Li *et al*. [79] demonstrated greater benefit of Tai Chi, as compared with stretching exercise, on sleep-quality outcomes, although this study did not include a non-treatment control group. Irwin *et al*. have found that Tai Chi Chih a westernized and standardized version of Tai Chi [80] induced significant improvements in sleep quality [81]. Finally, it is important to note that recent controlled trial data provide some promising evidence that Tai Chi may mitigate inflammation in older adults [82]. Likewise, other randomized controlled trials have documented reductions in proinflammatory cytokine activity following cognitive behavioural therapy [83] and meditation [84].

## 11. Conclusion

Restorative sleep is an important behavioural determinant of resilient aging. Successful aging is defined in part by the absence of medical and mental health morbidities or by reduced chronic medical burden in the face of chronic medical condition, which together are associated with adequate amount of sleep or good sleep. Conversely, when sleep wake activity is disrupted, successful adaptation to adverse challenges is compromised with increases in reporting of symptoms of pain, as well as depression. Sleep also contributes to the homeostatic regulation of inflammatory biology dynamics, with evidence that adequate sleep buffers age-related increases in inflammation, which likely translate into more positive health outcomes and lower risk of depression. Given these findings, efforts to promote healthy sleep habits and maintain sleep quality in aging have the potential to reduce inflammation and to build resilience in aging.

## Notes

The author does not report a conflict of interest and has nothing to disclose.
